# Building Topic-Driven Virtual IoTs in a Multiple IoTs Scenario

**DOI:** 10.3390/s19132956

**Published:** 2019-07-04

**Authors:** Paolo Lo Giudice, Antonino Nocera, Domenico Ursino, Luca Virgili

**Affiliations:** 1 Deloitte, Via Tortona, 25, 20144 Milano MI, Italy; 2 DIIES, University “Mediterranea” of Reggio Calabria, 80122 Reggio Calabria, Italy; 3 DIII, University of Pavia, 27100 Pavia, Italy; 4 DII, Polytechnic University of Marche, 60131 Ancona, Italy

**Keywords:** Internet of Things, Multiple IoTs, Profile of a Thing, topic-guided virtual IoTs, unsupervised and supervised approaches to virtual IoT construction

## Abstract

In the last years, several attempts to combine the Internet of Things (IoT) and social networking have been made. In the meantime, things involved in IoT are becoming increasingly sophisticated and intelligent, showing a behavior that tends to look like the one of users in social networks. Therefore, it is not out of place to talk about profiles of things and about information and topics exchanged among them. In such a context, constructing topic-driven virtual communities starting from the real ones operating in a Multi-IoT scenario is an extremely challenging issue. This paper aims at providing some contributions in this setting. First of all, it presents the concept of profile of a thing. Then, it introduces the concept of topic-guided virtual IoT. Finally, it illustrates two approaches (one supervised and one unsupervised) to constructing topic-guided virtual IoTs in a Multi-IoT scenario.

## 1. Introduction

The Internet of Things (hereafter, IoT) is currently considered the new frontier of the Internet. As a matter of fact, a lot of research results, along with the continuous emergence of increasingly challenging issues to address, can be found in the literature [1,2,3,4,5,6,7].

One of the most effective ways to represent and handle the IoT scenario leverages social networking paradigm [8]. In this direction, several social network-based approaches to modeling and managing IoTs have been presented in the literature. Three of the most advanced ones are the SIoT (Social Internet of Things) [9,10,11,12], the MIE (Multiple IoT Environment) [13] and the MIoT (Multiple IoTs) [14] paradigms. The MIoT paradigm is the last of these proposals; it aims at extending both SIoT and MIE in such a way as to preserve their strengths and avoid their weaknesses [14]. Roughly speaking, a MIoT can be seen as a set of related IoTs, i.e., as a set of related networks of things. Actually, a more precise definition of MIoT requires the introduction of the concept of instance of a thing in an IoT. Specifically, the instance of a thing in an IoT represents a virtual view of that thing in the IoT. The nodes associated with a thing in a MIoT represent the instances of the same thing in the different IoTs of the MIoT. Indeed, a thing can have several instances, one for each IoT which it participates to. The existence of more instances for one thing plays a key role in the MIoT paradigm because it allows the definition of cross relationships among the different IoTs.

We adopted the MIoT paradigm as the reference one in this paper. There are several reasons which justify this choice. Indeed:

- The MIoT paradigm, like the SIoT and the MIE ones, introduces the idea that objects can show a social behavior in the environment where they operate. This feature allows several advantages, like the possibility of resource sharing (see [10,11,12] for a comprehensive idea of these advantages).
- Differently from SIoT, which introduces a social behavior of objects but still models IoT as one huge network of objects extended worldwide, MIE, and much more MIoT, allow the “breakdown” of the whole huge IoT into multiple networks of smart objects interconnected with each other. This way to proceed is analogous to the evolution of social networking into social internetworking [15]. In particular, MIoT allows the management of situations in which the same object shows different behaviors in different networks it joined. Furthermore, MIoT makes an object to act as a bridge between two objects allowing them to communicate even if they belong to different networks and, therefore, are not directly connected with each other.

Another important trend characterizing the current IoT scenario regards the existence of increasingly sophisticated and intelligent things. These are becoming increasingly smart and social, as well as more and more capable of performing computations and storage on their own. Furthermore, they are increasingly connected to each other through more and more complex and sophisticated frameworks, often based on cloud and edge computing [10,11,12]. The new smart and social capabilities of things and of the environments handling their interoperability paves the way to a sort of “humanization” of things, i.e., to apply to things concepts and ideas typically considered prerogative of humans. One of them is certainly the presence of a profile of a thing. Indeed, if a thing interacts with other things and exchange data with them, it is possible to determine what are the most common concepts handled by it and, based on them, to construct a corresponding profile. Analogously to the profile of a human, the one of a thing depends on its past behavior and on the profile of the other things with which it interacts. As a consequence, it could be possible to think about both a content-based and a collaborative-filtering approach to handling thing profiles.

Furthermore, starting from the real IoTs of a MIoT, it is possible to construct virtual communities of things, based on common interests. Once again, this is an attempt to transfer behaviors typical of humans to things. As a matter of fact, in Social Network Analysis, it is well recognized that, accordingly to the homophily concept [16,17], humans tend to group together in communities sharing the same interests.

In the literature, a lot of efforts have been made to investigate human profiles and virtual communities of people, especially (but not only) in Social Network Analysis [18,19]. Instead, these topics have been little investigated in the Internet of Things.

In this paper, we aim at providing a contribution in this direction. First of all, we introduce the concept of profile of a thing. As the profile of a human, the one of a thing has two components. The former denotes its past behavior and can be used, for instance, to support content-based recommendations. The latter reflects its neighbors, i.e., the other things with which it most frequently comes into contact; it can be exploited, for instance, to support collaborative filtering recommendations.

After this, we introduce the concept of topic-guided virtual IoTs in a MIoT and we propose two approaches (one supervised and one unsupervised) to the construction of them in a MIoT. Differently from the real IoTs of a MIoT, which may encompass things with very heterogeneous profiles, topic-guided virtual IoTs should include all and only those things whose profile refers to specific topics. The supervised approach requires a user to provide a set of keywords of her interest. It aims at constructing a thematic IoT comprising all the keywords specified by the user. If such an IoT does not exists, it returns more thematic IoTs that, in the whole, comprise all the keywords specified by the user. She can choose whether to accept this set of virtual IoTs or to modify her query. The unsupervised approach tries to partition a MIoT into a set of virtual IoTs characterized by the maximum internal cohesion (in terms of topics present in the profiles of the corresponding things) and the minimum external coupling. Virtual IoTs in a MIoT provide a logic representation of the objects of a MIoT, which is not based on real links but on the content exchanged by them. As will be clear in the following, this can favor the effectiveness of information exchange, the construction of communities of objects (and, possibly, of the corresponding users) sharing the same interests and the suggestions of the objects most adequate to a given exigency.

This paper is organized as follows: in Section 2, we examine related literature. In Section 3, we provide an overview of the MIoT paradigm, because its comprehension is necessary to understand the rest of this paper. In Section 4, we introduce our definition of a thing’s profile. In Section 5, we propose our approaches to construct topic-guided virtual IoTs in a MIoT. In Section 6, we present our tests devoted to verify the performance of our approach. Finally, in Section 7, we draw our conclusions and have a look at future developments of our research efforts.

## 2. Related Literature

Since its introduction some years ago, the term “Internet of Things—IoT” has been associated with a huge variety of concepts, technologies and solutions [5,20,21,22]. In the latest years, with the advent of new technologies, such as big data and social networking, the very definition of this term is continuously changing. What IoT will become in the future depends on the evolution of these technologies [23] and their interaction with several other ones, such as Information Centric Networks [24,25,26,27,28,29,30] and Cloud [3,31,32]. As a matter of fact, the strengths of these last ones are exactly the features necessary to overcome the weaknesses of the current IoT concept [33]. Some examples of this combination can be already found in the literature [6,10,34,35].

The first attempts to apply social networking to the IoT domain can be found in [36,37,38,39]. In these papers, the authors propose to use human social network relationships to share services provided by a set of things. An important step forward is performed in [9], where the SIoT paradigm is introduced. Here, the authors propose an approach to creating relationships among things, without requiring the owner intervention. Thanks to this idea, things can autonomously crawl the network to find services and resources of their interest provided by other things. In [40], the same authors clearly highlight what are the main strengths of SIoT. Specifically: (i) the SIoT structure can be dynamically modified to ensure network navigability and to find new things; (ii) scalability is guaranteed, like in human social networks; (iii) a level of trustworthiness among things can be established; (iv) the past social network approaches can be redefined to solve problems typical of the IoT context [41].

One of the major drawbacks of the current IoT scenario is the presence of different technologies and solutions proposed by independent vendors to enable networking among objects. This poses the basis to a subsequent set of issues ranging from concept matching to technical compatibility, if heterogeneous smart-object-network solutions should be involved in the creation of a unique interoperable IoT [42,43]. In this research context, different works partially addressing and solving these problems have been proposed. Specifically, [44] presents a study on how ontologies and semantic data processing can be used to improve interoperability across heterogeneous IoT platforms. The authors consider two use cases, namely *Health Care* and *Trasportation and Logistics*, and, for each of them, provide a survey on the main ontologies available to describe and generalize concepts and relations.

In [45], instead, the authors focus their attention on the definition of a new framework for a fully functional mobile ad-hoc social network. In this paper, the term “mobile ad-hoc social network” refers to an IoT made of mobile devices. Of course, communication between this type of objects may happen in such a wide range of modes so that the referring scenario can be considered as a constellation of mobile networks interacting with each other. Concepts from real social networks are borrowed to define user profiles, which are built starting from the objects they own and the social network they belong to. One of the main contributions of this proposal is the definition of a profile-matching strategy based on semantics.

Another contribution in the context of interoperability is the one proposed in [46]. Here, the authors illustrate a novel architecture in which objects interact with each other by leveraging an open source cloud platform. The interaction among smart devices is information-and-service-driven and can be performed in both a centralized and a peer-to-peer mode. In [47], the authors propose *Acrost*, a system capable of retrieving data spread among heterogeneous IoT platforms by leveraging topics and semantics awareness. To build the metadata, Acrost uses two methodologies: the former exploits regular expression-based approaches, whereas the latter makes use of random fields-based strategies.

In order to address the issues arising when the interoperability among heterogeneous IoTs must be guaranteed, another research line proposes the extension of the results concerning Social Internetworking [15,48] (instead of social networking) to the Internet of Things. By following this strategy, the MIE (Multiple IoT Environment) [13] and the MIoT (Multiple IoTs) [14] paradigms have been proposed. As specified in the Introduction, this last paradigm is the reference one for this paper.

In [49], the authors present an approach to constructing a virtual data mart on which several knowledge discovery tasks can be performed. Clearly the kinds of virtual source constructed in the approach of [49] and in our own are very different. However, the general ideas underlying the two approaches are similar.

In the past, a lot of efforts have been made to investigate human profiles and virtual communities of people, especially (but not only) in Social Network Analysis ([18,19] provide two surveys about these topics). Instead, these issues have been little investigated in the Internet of Things. Specifically, to the best of our knowledge, a comprehensive, high-level abstraction approach to building and managing a profile of a thing, which also takes into account the content it exchanges during its interactions with other things, has not yet been proposed. Instead, some approaches focusing on community detection in IoT have been presented in the very recent literature. Even if they are very different (both in their purposes and in their ways proceed) from the ones of our approach, in the following we present an overview of some of them.

The approach of [50] uses structural information derived from the complex graph of an IoT to extract communities. It exploits a neighbor-based strategy to detect also overlapping communities. The approach of [51] uses data produced by sensors to define a multi-dimensional clustering. The obtained clusters are then mapped to communities of nodes in the original IoT network. To cope with the size of the data graph, the authors leverage state-of-the-art community detection approaches. Finally, they present a new community detection approach that enhances the Girvan-Newman algorithm by using hyperbolic network embedding.

Other works, instead, use knowledge from social networks to refine their results. A similar method is proposed in [52], even though here the strategy works in the opposite way. In fact, first communities are derived from structural information of owners’ social networks and, then, objects are seen as resources available inside each community.

Finally, the authors of [53] propose a new community detection algorithm working in a Social Internet of Things (SIoT) scenario. To achieve their objective, they make use of three metrics, namely social similarity, preference similarity and movement similarity. Social similarity is defined according to the concept of cooperativeness and community interest proposed in [54]. Preference similarity takes into account resource and service preferences of the involved things in the network. Finally, movement similarity specifies how much and how long two or more nodes are spatially close.

In [55], the authors propose a community detection approach working on an architecture capable of integrating the Internet of Things and social networking. This approach assumes that two nodes belong to the same community only if they are at most one hop apart and have at least two mutual friends. In order to construct communities, it exploits graph mining techniques.

As a consequence, it does not consider semantics and contents, but leverages only on network structure.

## 3. The MIoT Paradigm

In this section, we provide an overview of the MIoT paradigm, described in detail in [14], because it is the reference one for our definitions of virtual IoTs in a MIoT.

A MIoT M consists of a set of *m* Internets of Things. Formally speaking:

(1) $$M=\{I_{1},I_{2},\cdots ,I_{m}\}$$

where $I_{k}$ is an IoT.

Let $o_{j}$ be an object of M. We assume that, if $o_{j}$ belongs to $I_{k}$, it has an instance $\iota_{j_{k}}$, representing it in $I_{k}$. The instance $\iota_{j_{k}}$ consists of a virtual view (or, better, a virtual agent) representing $o_{j}$ in $I_{k}$. For example, it provides all the other instances of $I_{k}$, and the users who interact with $I_{k}$, with all the necessary information about $o_{j}$. Information stored in $\iota_{j_{k}}$ is represented according to the format and the conventions adopted in $I_{k}$.

A MIoT M can be represented by means of a graph-based notation. In particular, each IoT $I_{k}\in M$ can be modeled by means of a graph $G_{k}=\langle N_{k},A_{k}\rangle$. In this case:

- $N_{k}$ is the set of the nodes of $G_{k}$; there is a node $n_{j_{k}}$ for each instance $\iota_{j_{k}}\in I_{k}$, and vice versa.
- $A_{k}$ is the set of the arcs of $G_{k}$; there is an arc $a_{jq_{k}}=\left(n_{j_{k}},n_{q_{k}}\right)$ if there exists a physical link from $n_{j_{k}}$ to $n_{q_{k}}$.

Finally:

(2) M=〈N,A〉

Here:

(3) $$N=\overset{m}{\underset{k=1}{⋃}}N_{k};$$

(4) $$A=A_{I}\cup A_{C},$$

where

(5) $$A_{I}=\overset{m}{\underset{k=1}{⋃}}A_{k}$$

and

(6) $$A_{C}=\left\{\left(n_{j_{k}},n_{j_{q}}\right)|n_{j_{k}}\in N_{k},n_{j_{q}}\in N_{q},k\neq q\right\}.$$

$A_{I}$ is the set of the inner arcs (hereafter, *i-arcs*) of M; they relate instances (of different objects) belonging to the same IoT. $A_{C}$ is the set of the cross arcs (hereafter, *c-arcs*) of M; they relate instances of the same object belonging to different IoTs.

The description of the MIoT paradigm presented above highlights that it is possible to model a MIoT at two abstraction levels. The former represents a MIoT as a network and exploits concepts typical of this environment (such as nodes, arcs and so on). The latter models a MIoT as a set of IoTs and makes use of concepts closer to this scenario (such as instances, objects and so forth). Clearly, these two representations are simply two viewpoints of the same environment, and the concepts adopted by them can be used interchangeably. For example, there is a biunivocal correspondence between a node and an instance. However, in the reality, there are some cases in which it is better to use the concept of a node (for example, when we discuss about paths in a network—see below), whereas there are other situations in which it is better the use of the concept of instance (for example, when we discuss about the transactions carried out by two smart objects).

Furthermore, in a MIoT context, *a set $MD_{j}$ of metadata* can be associated with an object $o_{j}$. Our metadata model refers to the one of the IPSO (Internet Protocol for Smart Objects) Alliance [56]. Specifically $MD_{j}$ consists of three subsets, namely: *(i)*$MD_{j}^{D}$, i.e., the set of *descriptive metadata*; *(ii)*$MD_{j}^{T}$, i.e., the set of *technical metadata*; *(iii)*$MD_{j}^{B}$, i.e., the set of *behavioral metadata*. All details about these metadata can be found in [14].

## 4. Definition of a Thing’s Profile

In this section, we present our definition of a thing’s profile, which represents a first important contribution of this paper. As pointed out in the Introduction, analogously to what happens for human profiles, the profile of a thing can have two components. The former registers its past behavior and is extremely useful for content-based recommendations; for this reason, we call it “content-based component” in the following. The latter registers the main features of those things with which it mostly interacted in the past and can be used for collaborative filtering recommendations; for this reason, we call it “collaborative filtering component” in the following.

Before illustrating in detail the profile of a thing, we must introduce some preliminary concepts. First of all, given two instances $\iota_{j_{k}}$ of $o_{j}$ and $\iota_{q_{k}}$ of $o_{q}$ in $I_{k}$, we can define the set $tranSet_{jq_{k}}$ of the transactions from $\iota_{j_{k}}$ to $\iota_{q_{k}}$ as follows:

(7) $$tranSet_{jq_{k}}=\left\{T_{jq_{k_{1}}},T_{jq_{k_{2}}},\cdots ,T_{jq_{k_{v}}}\right\}$$

A transaction $T_{jq_{k_{t}}}\in tranSet_{jq_{k}}$ is represented as:

(8) $$T_{jq_{k_{t}}}=\langle reason_{jq_{k_{t}}},source_{jq_{k_{t}}},dest_{jq_{k_{t}}},start_{jq_{k_{t}}},finish_{jq_{k_{t}}},success_{jq_{k_{t}}},content_{jq_{k_{t}}}\rangle$$

Here:

- $reason_{jq_{k_{t}}}$ denotes the reason why $T_{jq_{k_{t}}}$ occurred, chosen among a set of predefined values.
- $source_{jq_{k_{t}}}$ indicates the starting node of the path followed by $T_{jq_{k_{t}}}$.
- $dest_{jq_{k_{t}}}$ represents the final node of the path followed by $T_{jq_{k_{t}}}$.
- $start_{jq_{k_{t}}}$ denotes the starting timestamp of $T_{jq_{k_{t}}}$.
- $finish_{jq_{k_{t}}}$ indicates the ending timestamp of $T_{jq_{k_{t}}}$.
- $success_{jq_{k_{t}}}$ denotes whether $T_{jq_{k_{t}}}$ was successful or not; it is set to true in the affirmative case, to false in the negative one, and to NULL if $T_{jq_{k_{t}}}$ is still in progress.
- $content_{jq_{k_{t}}}$ indicates the content “exchanged” from $\iota_{j_{k}}$ to $\iota_{q_{k}}$ during $T_{jq_{k_{t}}}$. In its turn, $content_{jq_{k_{t}}}$ presents the following structure:
  (9) $$content_{jq_{k_{t}}}=\langle format_{jq_{k_{t}}},fileName_{jq_{k_{t}}},size_{jq_{k_{t}}},topics_{jq_{k_{t}}}\rangle$$

Here:

- $format_{jq_{k_{t}}}$ indicates the format of the content exchanged during $T_{jq_{k_{t}}}$; the possible values are: “audio”, “video”, “image” and “text”.
- $fileName_{jq_{k_{t}}}$ denotes the name of the transmitted file.
- $size_{jq_{k_{t}}}$ indicates the size in bytes of the content.
- $topics_{jq_{k_{t}}}$ indicates the set of the content topics; it consists of a set of keywords representing the subjects exchanged during $T_{jq_{k_{t}}}$. It can be formalized as: $topics_{jq_{k_{t}}}=\left\{\left(kw_{jq_{k_{t}}}^{1},nkw_{jq_{k_{t}}}^{1}\right),\left(kw_{jq_{k_{t}}}^{2},nkw_{jq_{k_{t}}}^{2}\right),\ldots ,\left(kw_{jq_{k_{t}}}^{w},nkw_{jq_{k_{t}}}^{w}\right)\right\}$. In other words, the set of the topics of the $t^{th}$ transaction from $\iota_{j_{k}}$ to $\iota_{q_{k}}$ consists of *w* pairs; each pair consists of a keyword and the corresponding number of occurrences.

Now, we can define the set $tranSet_{j_{k}}$ of the transactions performed by $\iota_{j_{k}}$ in $I_{k}$. Specifically, let $Inst_{k}$ be the set of the instances of $I_{k}$. Then:

(10) $$tranSet_{j_{k}}=\underset{\iota_{q_{k}}\in Inst_{k},\iota_{q_{k}}\neq \iota_{j_{k}}}{⋃}tranSet_{jq_{k}}$$

In other words, the set $tranSet_{j_{k}}$ of the transactions performed by an instance $\iota_{j_{k}}$ is given by the union of the sets of the transactions from $\iota_{j_{k}}$ to all the other instances of $I_{k}$.

After having defined $tranSet_{j_{k}}$, we must introduce the following operators:

- ⊎: it receives a set $\left\{entitySet_{1},entitySet_{2},\cdots ,entitySet_{t}\right\}$ of entity sets and performs their union not eliminating the duplicates but reporting the number of their occurrences. Therefore, this operator returns a set of pairs $\left\{\left(entity_{1},ne_{1}\right),\left(entity_{2},ne_{2}\right),\cdots ,\left(entity_{w},ne_{w}\right)\right\}$ in which the pair $\left(entity_{r},ne_{r}\right)$ indicates the $r^{th}$ entity and the number of its occurrences. In counting it, ⊎ takes the presence of synonymies and homonymies into account. These properties can be computed (for terms, images, etc.) by applying the classical approaches proposed in the past literature [57,58].
- avgFileSize: it receives a set of files and computes their average size.

We are now able to define the profile $P_{jq_{k}}$ of the relationship existing between two instances $\iota_{j_{k}}$ and $\iota_{q_{k}}$, which performed a set $tranSet_{jq_{k}}=\left\{T_{jq_{k_{1}}},T_{jq_{k_{2}}},\cdots ,T_{jq_{k_{v}}}\right\}$ of transactions. As we will see in the following, this profile plays a crucial role in the definition of the content-based component of a thing’s profile and is indirectly used also in the definition of the collaborative filtering component of it. Specifically:

(11) $$\begin{array}{c} P_{jq_{k}}=\langle reasonSet_{jq_{k}},sourceSet_{jq_{k}},destSet_{jq_{k}},avgSzAudio_{jq_{k}},avgSzVideo_{jq_{k}},avgSzImage_{jq_{k}},\\ avgSzText_{jq_{k}},successFraction_{jq_{k}},topicSet_{jq_{k}}\rangle \end{array}$$

where:

- $reasonSet_{jq_{k}}={⊎}_{t=1..v}\left(reason_{jq_{k_{t}}}\right)$;
- $sourceSet_{jq_{k}}={⊎}_{t=1..v}\left(source_{jq_{k_{t}}}\right)$;
- $destSet_{jq_{k}}={⊎}_{t=1..v}\left(dest_{jq_{k_{t}}}\right)$;
- $avgSzAudio_{jq_{k}}=AvgFileSize_{t=1..v}\left\{fileName_{jq_{k_{t}}}|format_{jq_{k_{t}}}=‘‘audio"\right\}$;
- $avgSzVideo_{jq_{k}}=AvgFileSize_{t=1..v}\left\{fileName_{jq_{k_{t}}}|format_{jq_{k_{t}}}=‘‘video"\right\}$;
- $avgSzImage_{jq_{k}}=AvgFileSize_{t=1..v}\left\{fileName_{jq_{k_{t}}}|format_{jq_{k_{t}}}=‘‘image"\right\}$;
- $avgSzText_{jq_{k}}=AvgFileSize_{t=1..v}\left\{fileName_{jq_{k_{t}}}|format_{jq_{k_{t}}}=‘‘text"\right\}$;
- $successFraction_{jq_{k}}=\frac{\left|\{T_{jq_{k_{t}}}|T_{jq_{k_{t}}}\in tranSet_{jq_{k}},success_{jq_{k_{t}}}=true\}\right|}{v}$;
- $topicSet_{jq_{k}}={⊎}_{t=1..v}\left(topics_{jq_{k_{t}}}\right)$.

If we introduce the operator ⨆, which compactly represents the set of operations for obtaining a profile of a pair of instances $P_{jq_{k}}$ starting from the corresponding transactions, we can formalize the previous tasks by means of only one operation as follows:

(12) $$P_{jq_{k}}=\underset{t=1..v}{⨆}T_{jq_{k_{t}}}$$

Now, let $\iota_{j_{k}}$ be the instance of the object $o_{j}$ in the IoT $I_{k}$. Let $Inst_{j_{k}}$ be the set of the instances of $I_{k}$ with which $\iota_{j_{k}}$ performed at least one transaction in the past. In this case, we can define the content-based component of the profile $P_{j_{k}}$ of $\iota_{j_{k}}$ as:

(13) $$P_{j_{k}}=\underset{\iota_{q_{k}}\in Inst_{j_{k}}}{⨆}P_{jq_{k}}$$

Finally, let $o_{j}$ be an object and let $\left\{I_{1},I_{2},\cdots ,I_{l}\right\}$ be the set of the IoTs which it participates to. Let $ObjInst_{j}$ be the instances of $o_{j}$ in the IoTs of the MIoT. We can define the content-based component of the profile $P_{j}$ of $o_{j}$ as:

(14) $$P_{j}=\underset{\iota_{j_{k}}\in ObjInst_{j}}{⨆}P_{j_{k}}$$

After having defined the content-based component of an instance and an object, in order to present the corresponding collaborative filtering components, we must introduce the concept of neighborhoods of an instance $\iota_{j_{k}}$ in an IoT $I_{k}$. Specifically, the structural neighborhood $sNbh(\iota_{j_{k}})$ of $\iota_{j_{k}}$ is defined as:

(15) $$sNbh\left(\iota_{j_{k}}\right)=sNbh^{out}\left(\iota_{j_{k}}\right)\cup sNbh^{in}\left(\iota_{j_{k}}\right)$$

where:

(16) $$sNbh^{out}\left(\iota_{j_{k}}\right)=\left\{\iota_{q_{k}}|\left(n_{j_{k}},n_{q_{k}}\right)\in A_{I}\right\}$$

(17) $$sNbh^{in}\left(\iota_{j_{k}}\right)=\left\{\iota_{q_{k}}|\left(n_{q_{k}},n_{j_{k}}\right)\in A_{I}\right\}$$

Furthermore, we can also define the behavioral neighborhood $bNbh(\iota_{j_{k}})$ of $\iota_{j_{k}}$ as:

(18) $$bNbh\left(\iota_{j_{k}}\right)=bNbh^{out}\left(\iota_{j_{k}}\right)\cup bNbh^{in}\left(\iota_{j_{k}}\right)$$

where:

(19) $$bNbh^{out}\left(\iota_{j_{k}}\right)=\{\iota_{q_{k}}|\iota_{q_{k}}\in sNbh^{out}\left(\iota_{j_{k}}\right),|tranSet_{jq_{k}}\left|>0\right\}$$

(20) $$bNbh^{in}\left(\iota_{j_{k}}\right)=\{\iota_{q_{k}}|\iota_{q_{k}}\in sNbh^{in}\left(\iota_{j_{k}}\right),|tranSet_{qj_{k}}\left|>0\right\}$$

In other words, $bNbh(\iota_{j_{k}})$ consists of those instances directly connected to $\iota_{j_{k}}$ from the structural viewpoint that shared at least one transaction with $\iota_{j_{k}}$.

We are now able to present the collaborative filtering component $P_{j_{k}}^{\prime }$ of the profile of an instance $\iota_{j_{k}}$ in $I_{k}$. It can be defined as follows:

(21) $$P_{j_{k}}^{\prime }=\underset{\iota_{q_{k}}\in bNbh\left(\iota_{j_{k}}\right)}{⨆}\left(P_{q_{k}}⊔{P^{\prime }}_{q_{k}}\right)$$

Clearly, this definition is recursive and an accurate computation would require the resolution of a system with a number of equations and variables equal to the number of instances. In real situations, as there could be thousands or millions of instances in a MIoT, the time necessary to solve this system may easily become unacceptable. As a consequence, it appears reasonable to consider an approximate definition of $P_{q_{k}}$ that is much simpler to handle. It is formalized as:

(22) $$P_{j_{k}}^{\prime }=\underset{\iota_{q_{k}}\in bNbh\left(\iota_{j_{k}}\right)}{⨆}P_{q_{k}}$$

After having introduced the two components of the profile of an instance $\iota_{j_{k}}$ of $I_{k}$, we can combine them for defining the overall profile $\bar{P_{j_{k}}}$ of $\iota_{j_{k}}$. It is defined as the union of the profiles $P_{j_{k}}$ and $P_{j_{k}}^{\prime }$ performed by means of the operator ⊔:

(23) $$\bar{P_{j_{k}}}=P_{j_{k}}⊔P_{j_{k}}^{\prime }$$

Finally, we can define the overall profile of an object $o_{j}$ as follows:

(24) $$\bar{P_{j}}=\underset{k=1..l}{⨆}\bar{P_{j_{k}}}$$

## 5. Topic-Guided Virtual IoTs in a MIoT and Approaches to Constructing Them

In this section, we present a supervised and an unsupervised approach to constructing topic-guided virtual IoTs in a MIoT.

### 5.1. Supervised Approach

The supervised approach for the construction of topic-guided virtual IoTs in a MIoT requires the user to specify a query *Q* consisting of some keywords of her interest. It tries to construct a thematic virtual IoT in such a way that each of its instances contains at least one keyword of *Q* in the content-based component of its profile. If such a virtual IoT does not exist, our approach returns a minimal set of thematic IoTs that, on the whole, contain, in the content-based component of the profile of their instances, all the keywords specified by the user. In this last case, she can choose whether to accept this set of IoTs or modify her query.

Before describing in detail this approach, we must introduce a new operator $J^{*}$ that represents a modified Jaccard coefficient, as we will see below.

$J^{*}$ receives two sets of topics (We recall that, in our context, a topic is a pair (kw,nkw), where kw is a keyword and nkw is the corresponding number of occurrences.) $topicSet=\{\left(kw_{1},nkw_{1}\right),\left(kw_{2},nkw_{2}\right),\cdots ,\left(kw_{p},nkw_{p}\right)\}$ and $topicSet^{\prime }=\left\{\left(kw_{1}^{\prime },nkw_{1}^{\prime }\right),\left(kw_{2}^{\prime },nkw_{2}^{\prime }\right),\cdots ,\left(kw_{p}^{\prime },nkw_{p}^{\prime }\right)\right\}$ and computes the Jaccard coefficient between them. In carrying out this task, it considers the number of occurrences of each keyword and its possible synonyms.

More formally, first it computes the set:

(25) $$\begin{array}{c} commonTS=\{\left(kw,nkw+nkw^{\prime }\right)|\left(kw,nkw\right)\in topicSet,\left(kw^{\prime },nkw^{\prime }\right)\in topicSet^{\prime },\\ kw\;\mathrm{is}\;\mathrm{identical}\;\mathrm{to}\;\mathrm{or}\;\mathrm{synonymous}\;\mathrm{of}\;kw^{\prime }\} \end{array}$$

Then, it computes the final result as:

(26) $$J^{*}\left(topicSet,topicSet^{\prime }\right)=\frac{\sum_{(kw,nkw)\in commonTS}nkw}{\sum_{(kw,nkw)\in topicSet}nkw+\sum_{\left(kw^{\prime },nkw^{\prime }\right)\in topicSet^{\prime }}nkw^{\prime }}$$

After having introduced $J^{*}$, we can describe our approach. Specifically:

- It starts when a user specifies a query *Q* consisting of *r* keywords:
  (27) $$Q=\{kw_{1},kw_{2},\cdots ,kw_{r}\}$$
  It searches for all the instances of the MIoT having at least one topic whose keyword is identical to, or synonymous of, at least one keyword specified in *Q*. These instances, as a whole, represent the set of candidate instances to be included in the new thematic view. We call this set CI (Candidate Instances).
- However, the fact that an instance ι∈CI has a keyword in common with *Q* is necessary but not sufficient for it to be chosen. In fact, it is advisable that ι has more keywords in common with *Q* and, possibly, that the common keywords are among the ones of ι with the highest number of occurrences. This condition can be guaranteed by the usage of the operator $J^{*}$.
  In particular, our approach first constructs $Q^{\prime }=\left\{\left(kw,1\right)|kw\in Q\right\}$ in such a way as to make the application of $J^{*}$ on the keywords specified by the user possible. Then, it constructs the set RI (Real Instances) of those instances of CI whose topics have a significant similarity with the keywords of *Q*:
  (28) $$\mathrm{RI}=\{\iota \in \mathrm{CI}|J^{*}\left(topicSet_{\iota },Q^{\prime }\right)>th_{J}\}$$
  Here, $th_{J}$ is a suitable tuning threshold.
- Now, our approach can start to construct the thematic view $V_{Q}$ corresponding to *Q*.
  - It first creates a node $n_{\iota }$ in $V_{Q}$ for each instance ι of RI. Let $n_{\iota_{1}}$ and $n_{\iota_{2}}$ be the nodes corresponding to two instances $\iota_{1}$ and $\iota_{2}$ belonging to RI.
    * If an i-arc exists between the nodes corresponding to $\iota_{1}$ and $\iota_{2}$ in the MIoT M, then an i-arc is also created between the nodes $n_{\iota_{1}}$ and $n_{\iota_{2}}$ in $V_{Q}$.
    * Instead, if a c-arc exists between the nodes corresponding to $\iota_{1}$ and $\iota_{2}$ in M, then $n_{\iota_{1}}$ and $n_{\iota_{2}}$ are merged in a unique node $n_{\iota_{12}}$ in $V_{Q}$. This task is motivated by the fact that $n_{\iota_{1}}$ and $n_{\iota_{2}}$ represent different instances of the same object in different real IoTs, but they represent the same instance in the same virtual IoT; as a consequence, they must be merged and no cross arc can exist between them. The profile $\bar{P_{12}}$ of $n_{\iota_{12}}$ is obtained by applying the operator ⨆ on the profiles $\bar{P_{1}}$ of $\iota_{1}$ and $\bar{P_{2}}$ of $\iota_{2}$.
- Finally, our approach adds a disconnected node in $V_{Q}$ for each keyword in *Q* such that there is no MIoT instance having at least one topic whose keyword is identical to, or synonymous of, it (The rationale underlying this step will be clearer in the following.).
- At this point, two cases may occur. In particular:
  - It could happen that $V_{Q}$ is connected. In this case, it is returned as the answer to the query *Q* submitted by the user.
  - If $V_{Q}$ is not connected and if the number of its connected components is less than a certain threshold, our approach adds the minimum number of “fictitious” i-arcs necessary to make $V_{Q}$ connected.
  - Otherwise, if the number of connected components of $V_{Q}$ is higher than a certain threshold, our approach concludes that a unique thematic virtual IoT corresponding to the keywords specified by the user does not exist and returns the thematic views related to the connected components of $V_{Q}$. At this point, the user can decide whether to accept these thematic views or to modify the query in such a way as to construct a unique thematic view by re-applying all the above mentioned steps starting from the new query.

### 5.2. Unsupervised Approach

The unsupervised approach begins with the construction of a support network N starting from the MIoT M. In particular:

- For each node $n_{\iota_{k}}$ of M, a node $\bar{n_{\iota_{k}}}$ is added in N.
- For each i-arc $\left(n_{\iota_{j_{k}}},n_{\iota_{q_{k}}}\right)$ in M, an (unoriented) arc $\left(\bar{n_{\iota_{j_{k}}}},\bar{n_{\iota_{q_{k}}}}\right)$ is added in N. The arcs of N are weighted. The weight of the arc $\left(\bar{n_{\iota_{j_{k}}}},\bar{n_{\iota_{q_{k}}}}\right)$ is obtained by applying the operator $J^{*}$ on the topic sets $topicSet_{j_{k}}$ and $topicSet_{q_{k}}$ of $\iota_{j_{k}}$ and $\iota_{q_{k}}$, respectively. Therefore, the weight of an arc in N belongs to the real interval [0,1]; the higher this weight the higher the semantic similarity between the topics of the profiles $\bar{P_{j_{k}}}$ and $\bar{P_{q_{k}}}$ of $\iota_{j_{k}}$ and $\iota_{q_{k}}$, respectively.
- For each c-arc in M, which relates two instances $n_{\iota_{j_{k}}}$ and $n_{\iota_{j_{q}}}$ of the same object $o_{j}$ in two different IoTs $I_{k}$ and $I_{q}$, the two nodes $\bar{n_{\iota_{j_{k}}}}$ and $\bar{n_{\iota_{j_{q}}}}$ in N, corresponding to the nodes $n_{\iota_{j_{k}}}$ and $n_{\iota_{j_{q}}}$ in M, are merged into a unique node $\bar{n_{\iota_{j}}}$. This node inherits all the arcs of $\bar{n_{\iota_{j_{k}}}}$ and $\bar{n_{\iota_{j_{q}}}}$.

At the end of these steps, it could happen that two or more arcs relate the same nodes $\bar{n}$ and $\bar{n^{\prime }}$ in N. In this case, all these arcs must be merged into a single arc. Clearly, it is necessary to determine the weight of this arc. Here, it appears reasonable that it must be higher than or equal to the maximum weight of the merged arcs. To reach this objective, our approach operates as follows. Let $\{\left(\bar{n},\bar{n^{\prime }},\bar{w^{1}}\right),\left(\bar{n},\bar{n^{\prime }},\bar{w^{2}}\right),$$\cdots ,(\bar{n},\bar{n^{\prime }},\bar{w^{s}})\}$ be the arcs to merge, ordered by decreasing weight. The new arc $\left(\bar{n},\bar{n^{\prime }},\bar{w}\right)$ will have a weight equal to:

(29) $$\bar{w}=min\left(1,\bar{w_{1}}+\alpha \sum_{k=2..s}\bar{w_{k}}\right)$$

In other words, in the computation of $\bar{w}$, the arcs with the maximum weight will contribute with all their weight. All the other arcs will contribute to a lesser extent, with a fraction of their weight. This last is determined by means of the coefficient α.

Once the construction of N has been completed, the thematic views are derived by applying on N a graph clustering algorithm among the ones already existing in the literature (see [59] for a survey on them).

### 5.3. Discussion

An important issue about the supervised and the unsupervised approaches to address regards their scalability or, better, the possibility to use them in MIoTs comprising thousands or even millions of nodes.

With regard to this issue, first of all we observe that both approaches aim at deriving virtual IoTs which are, then, exploited by users to perform their desired tasks (such as querying). As a consequence, we can distinguish two moments in the life of a MIoT, namely: *(i)* the construction of virtual IoTs, which can be performed *offline*, and *(ii)* their usage, which is generally carried out *online*.

The first moment is computationally expensive because it involves several network operations in the supervised approach and a clustering activity in the unsupervised one. Clustering’s computational cost is intrinsically exponential even if all the corresponding methods adopted in the reality are heuristic and most of them have a linear or a quadratic computational complexity. In any case, as pointed above, this task is performed offline and rarely because it is necessary only when many changes have been made in the MIoT.

The second moment is certainly less expensive; its cost depends on the size of the involved clusters; in fact, each user activity generally involves one or a few clusters. Concerning this aspect, it is important to verify: *(i)* if clustering is possible in presence of huge MIoTs, and *(ii)* how the size of clusters increases against the growth of the MIoT. As for the first point, we observe that, in the past, several algorithms have been specifically conceived to cluster a huge amount of elements [60]. Concerning the second point, instead, first we observe that the size of clusters can be determined by suitably tuning the parameters of the selected clustering algorithm. However, it could be interesting to verify how much the size of clusters increases if we maintain constant all the clustering algorithm parameters and the MIoT size increases. We decided to perform this experiment. It is described in detail in Section 6.6. Here, we evidence the obtained results, i.e., that when the MIoT size highly increases, the cluster size slightly grows, whereas the number of clusters increases very much. This is a positive result for our purposes because the parameter to monitor for investigating the performance obtained during the second moment is just cluster size.

Another important issue to investigate regards the possible existence of a unique framework handling all the objects of the MIoT and, therefore, in principle, thousands or millions of objects. With regard to this aspect, we evidence that, in the past, several attempts have been successfully performed in this direction (think, for instance, of the SIoT framework proposed in [9,40]). Clearly, we understand that, in the future, the number of objects possibly belonging to a MIoT is enormously higher than the number of objects available in the past IoT frameworks. However, we point out that: *(i)* our approach needs to store only the metadata of the involved objects, and these are small; *(ii)* the real objects can operate in a distributed environment thanks to the new available technologies, such as cloud, edge and fog computing, which can ease the organization and the management of distributed contexts.

## 6. Experiments

In this section, we present the experimental campaign that we carried out to evaluate the performance of our approach from several viewpoints. Specifically, we describe our dataset in a subsection, whereas, in the next ones, we illustrate our tests, along with the underlying motivations and the obtained results.

### 6.1. Adopted Dataset

To perform our experiments, we had the necessity to create several MIoTs with different sizes, ranging from hundreds to thousands of nodes. Since, currently, real MIoTs with the size and the variety handled by our model do not exist yet, we had to realize a MIoT simulator, i.e., a tool that, starting from real data, is capable of simulating MIoTs with certain characteristics specified by the user.

The MIoTs created by our simulator follow the model described in Section 3. In order to perform its task, our simulator carries out the following steps: *(i)* creation of objects; *(ii)* creation of object instances; *(iii)* creation of instance connections; *(iv)* creation of instance profiles.

Our MIoT simulator is also provided with a suitable interface allowing a user to “personalize” the MIoT to construct by specifying the desired values for several parameters, such as the number of nodes, the maximum number of instances of an object, and so forth.

To make “concrete” and “plausible” the created MIoT, our simulator leverages a real dataset. It regards the taxi routes in the city of Porto from 1 July 2013 to 30 June 2014. It can be found at the address http://www.geolink.pt/ecmlpkdd2015-challenge/dataset.html. Each route contains several Points of Interests corresponding to the GPS coordinates of the vehicle.

We partitioned the city of Porto in six areas and associated a real IoT with each of them. Our simulator associates an object with a given route recorded in the dataset and an object instance for each partition of a route belonging to an area. It creates a MIoT node for each instance and a c-arc for each pair of instances belonging to the same route. Furthermore, it creates an i-arc between two nodes of the same IoT if the length of the time interval between the corresponding routes is less than a certain threshold $th_{t}$. The weight of the i-arc indicates the length of this time interval. The value of $th_{t}$ can be specified through the constructor interface. Clearly, the higher $th_{t}$ the more connected the constructed MIoT.

As far as instance profiles are concerned, since there are no thing profiles available (indeed, the concept of thing profile is one of the main novelties introduced in this paper), we had to simulate them. However, we aimed to make them as real as possible. In order to increase the likelihood of constructed MIoTs, we performed a sentiment analysis task for each of the six areas in which we partitioned the city of Porto and for each day which the dataset refers to. For this purpose, we leveraged IBM Watson on the social media and blogs it uses as default. Having this data at disposal, our simulator assigns to each instance the most common topics (along with the corresponding occurrences) discussed in that area in the day on which the corresponding route took place. The constructed MIoTs are returned in a format that can be directly processed by the cypher-shell of Neo4J (see below).

Some features of the constructed MIoTs are reported in Table 1. The interested reader can find the MIoTs adopted in the experiments described in this section at the address http://daisy.dii.univpm.it/miot/datasets/virtualIoTs.

We carried out all the tests presented in this section on a server equipped with an Intel I7 Quad Core 7700 HQ processor and 16 GB of RAM with Ubuntu 16.04 operating system.

To implement our approaches we adopted:

- Python, powered with the NetworkX library, as programming language;
- Neo4J (Version 3.4.5) as underlying DBMS; we also exploited some plugins of Neo4J to perform community detection and to compute clustering coefficients.

### 6.2. Cohesion of the Obtained Topic-Guided Virtual IoTs

Our first test started from the idea that if our approach aims at extracting virtual thematic IoTs, they should present both a structural and a semantic cohesion higher than the corresponding ones characterizing the original IoTs of the MIoT. This experiment was devoted to evaluate if this assumption is verified. We considered two well known structural cohesion parameters used in network analysis literature, namely *clustering coefficient* and *density* [61]. Both of them range in the real interval [0,1]; the higher their value the higher the corresponding network cohesion. In the following, first we test the supervised approach and, then, we consider the unsupervised one.

#### 6.2.1. Supervised Approach

In this test, we run our supervised approach on ten MIoTs, $M_{1}$, ⋯, $M_{10}$, consisting of 176, 301, 485, 778, 946, 1256, 1725, 2028, 3544 and 5024 nodes. Clearly, the number of IoTs for each MIoT was equal to six, one for each area of the city of Porto that we have defined. For each MIoT, we submitted a set of 10 queries consisting of 1 (resp., 2, 4, 6, 8 and 10) word(s).

Each query returned a virtual thematic IoT for which we computed the corresponding clustering coefficient and density. Finally, we averaged the obtained results for each MIoT and for each set of queries, and we compared them with the average clustering coefficient and the average density of the corresponding real IoTs. The obtained results are reported in Table 2 and Table 3.

From the analysis of these tables, we can observe that, in almost all circumstances, the values of both clustering coefficient and density are higher or much higher for the virtual thematic IoTs than for the real ones. This is clearly a confirmation of the goodness of our supervised approach, which returns topic-guided IoTs more cohesive than the original ones. We also observe that when |Q| increases, the values of both clustering coefficient and density increases. This can be explained by observing that, in processing *Q*, our approach takes the portions of networks containing at least one keyword of *Q*. When |Q| increases, the portion of networks selected by our approach increases too, and the probability of selecting a very high number of edges (i.e., a number so high to lead to an increase of clustering coefficient and density) increases as well.

#### 6.2.2. Unsupervised Approach

In this test, we run our unsupervised approach, powered with the Louvain graph clustering algorithm [62] as underlying engine, on the same MIoTs described in Section 6.2.1. For each MIoT, we computed the average clustering coefficient and the average density of real and virtual IoTs. The obtained results are reported in Table 4.

From the analysis of this table we can observe that, in this case, analogously to what happened for the supervised approach, the cohesion level of the virtual IoTs is higher or much higher than the corresponding ones of the real original IoTs. Interestingly, both clustering coefficient and density values obtained by the unsupervised approach are generally higher than those returned by the supervised one, at least when the MIoT size is small. Instead, when the MIoT size is large, they become lower than the ones of the supervised approach. Actually, the increase of both clustering coefficient and density when the MIoT size increases is significant for the supervised approach, whereas it is more limited for the unsupervised one.

### 6.3. Average Fraction of Merged C-Nodes and Analysis of Node Distribution in Virtual IoTs

Another quality parameter for virtual IoTs returned by our approach regards the average number of merged c-nodes present in each of them. Indeed, the presence of merged c-nodes in an IoT is an indicator of the fact that this IoT is capable of connecting concepts coming from different real IoTs, and, therefore, from concepts whose relationships would have been uncaptured otherwise, or, in other words, that the knowledge it is presenting is new and did not exist previously. Clearly, the higher the fraction of merged c-nodes and the higher the fraction of different original IoTs they belong to, the higher the connecting capability of virtual IoTs.

Also for this experiment, we considered the ten MIoTs described in Section 6.2 and performed the same tasks illustrated therein for both the supervised and the unsupervised approaches. The obtained results are reported in Table 5, Table 6 and Table 7.

From the analysis of these tables, we observe that both the supervised and the unsupervised approaches return satisfying results. As for the supervised approach, we can observe that the fraction of merged c-nodes increases when the size of MIoT increases. Furthermore, we can also observe a slight increase of this fraction when |Q| increases. The same trends can be observed for the average fraction of involved real IoTs, even if, for this parameter, its increase against the increase of |Q| is more pronounced. As for the unsupervised approach, we can observe that the average fraction of merged nodes is always very high, independently of the MIoT size. By contrast, in this case, the fraction of involved real IoTs is quite high even if lower than the ones generally observed for the supervised approach. Furthermore, its value does not significantly change when the MIoT size increases.

In order to deepen this investigation, for each virtual IoT, we compared the distribution of its nodes against the real IoTs they belong to. Indeed, if almost all the nodes of a virtual IoT derive from only one real IoT, the information contribution provided by the virtual IoT would be very small because it would be analogous to the one provided by the corresponding real IoT. By contrast, if the nodes of a virtual IoT homogeneously derive from several real IoTs, then the knowledge it provides is really new, and this knowledge would be uncaptured and lost if the new IoT had not been extracted. On the basis of this reasoning, we evaluated the heterogeneity of the provenance of the various nodes of each virtual IoT (see below). For this purpose, we adapted the Herfindahl Index [63] to our context. This index is very used in several research fields of Economics from several decades; for instance, it is exploited to evaluate the concentration degree in an industry.

In order to adapt the Herfindahl Index to our scenario, consider a MIoT M consisting of *s* real IoTs $(R_{1},R_{2},\cdots ,R_{s}$). Consider, also, a virtual IoT $V_{j}$ derived by either the supervised or the unsupervised approach. Let $n_{j}$ be the number of nodes of $V_{j}$ and let $\frac{n_{j_{k}}}{n_{j}}$, 1≤k≤s, be the fraction of the nodes of $V_{j}$ belonging to $R_{k}$ (i.e., the $k^{th}$ real IoT of the MIoT). The Herfindahl Index $H_{j}$ of $V_{j}$ is defined as $\sum_{k=1}^{s}{\left(\frac{n_{j_{k}}}{n_{j}}\right)}^{2}$. $H_{j}$ ranges in the real interval $\left[\frac{1}{s},1\right]$; the higher its value, the higher the concentration degree of the nodes of $R_{k}$ in $V_{j}$. Clearly, as previously pointed out, one property desired for our approach is the ability to construct virtual IoTs connecting nodes that belong to different real IoTs in such a way as to extract knowledge that would be lost otherwise. If we report this property to the Herfindahl Index, this implies to obtain a value of this index as lower as possible (Consider that, since we have six real IoTs in our MIoTs, the minimum value of the Herfindahl Index is $\frac{1}{6}=0.167$.).

We computed the average Herfindahl Index of the thematic IoTs returned by both the supervised and the unsupervised approaches by considering the ten MIoTs described in Section 6.2 and performing the same tasks illustrated therein. The obtained results are reported in Table 8 and Table 9.

These tables evidence that also the analysis based on object distribution and Herfindahl Index returns very satisfying results that confirm and strengthen those obtained by examining the average fraction of merged nodes involved in a virtual IoT. Interestingly, as for this parameter, we observe that the supervised approach returns excellent results, very close to the best ones. By contrast, the unsupervised approach returns good results, even if those returned by the supervised approach are better.

### 6.4. Computation Time

In this experiment, we aimed at evaluating the variation of the computation time of both the supervised and the unsupervised approaches against the variation of the size of the involved MIoT. Furthermore, as for the supervised approach, we also evaluated the variation of the computation time against the variation of the size of queries.

**Figure 1:**
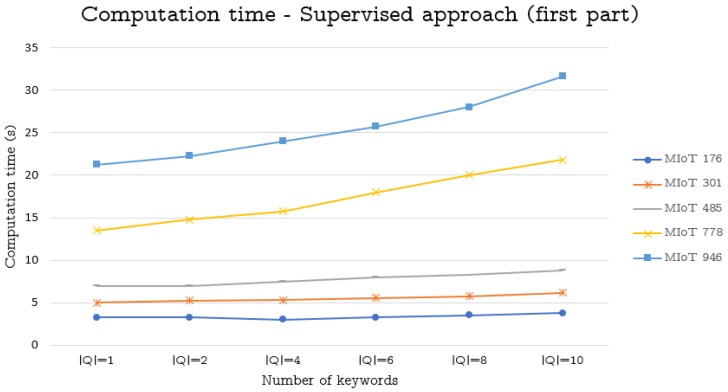
Computation time (in seconds) against the size of MIoTs and queries used to generate the virtual IoTs (supervised approach)—first part.

**Figure 2:**
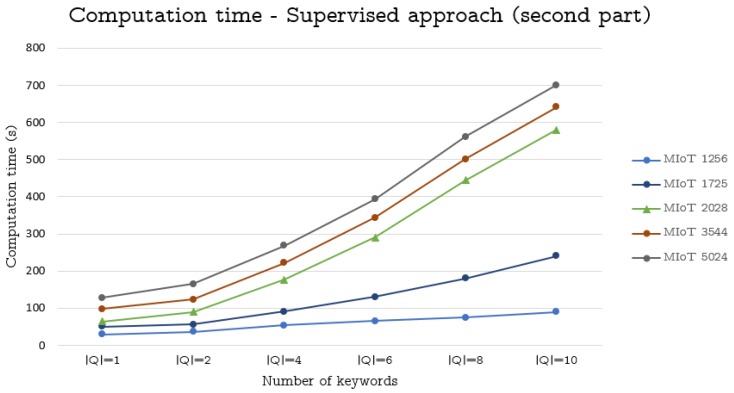
Computation time (in seconds) against the size of MIoTs and queries used to generate the virtual IoTs (supervised approach)—second part

**Figure 3:**
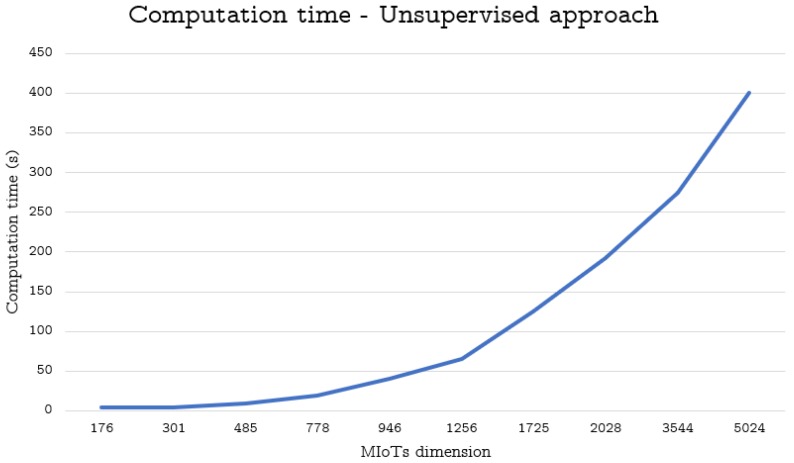
Computation time (in seconds) against the size of MIoTs (unsupervised approach)

To perform this task, we considered the ten MIoTs described in Section 6.2 and carried out the same tasks illustrated therein. Finally, we measured the corresponding average computation times. The obtained results are reported in Figure 1, Figure 2 and Figure 3.

From the analysis of these figures, we can observe that our approaches obtain satisfying results. Specifically, as for the supervised approach, the computation time is always very low for MIoTs having at most 1256 nodes. Instead, for MIoTs with more than 2028 nodes, the computation time is low for |Q|=1 or |Q|=2. Then, it increases, even if it remains acceptable for |Q|=4 and |Q|=6, whereas it becomes excessive for |Q|=8 and |Q|=10. However, with regard to this fact, we must point out that queries consisting of 8 or 10 keywords are very uncommon (It is worth pointing out that the topics considered by our approach for constructing a thing’s profile are extremely generic and heterogeneous. As a consequence, in our scenario, a query with 8 or 10 keywords would encompass a great number of different topics and, as such, it would not be generally able to capture a clear and specific desire of a user.).

As for the unsupervised approach, its computation time is still acceptable also for 2028 nodes. It starts to become excessive with MIoTs consisting of at least 10,000 nodes.

### 6.5. Our Approaches’ Capability of Improving the Efficiency of Information Dissemination

This experiment was devoted to measure the efficiency of both supervised and unsupervised approaches. The rationale underlying this experiment is that if some information must be transferred from a source object $o_{s}$ to a target one $o_{t}$, the number of objects to be contacted for this task should be minimized. At the same time, if an object is involved in an information dissemination task, it would be desiderable that the information it is transmitting is also useful for it (which, in our case, means that it is in line with the interests of its profile).

In order to perform this experiment, we randomly selected some pairs of (source, target) nodes from our MIoT. Let $\left(n_{s},n_{t}\right)$ be one of these pairs. We verified if there existed at least one virtual IoT comprising both $n_{s}$ and $n_{t}$ (This is always true for the unsupervised approach, whereas it could not happen for the supervised one.). In the negative case, we discarded that pair. Let V be a virtual IoT comprising both $n_{s}$ and $n_{t}$.

After this, we computed the number $num_{st}^{V}$ (resp., $\hat{num_{st}^{V}}$) of MIoT nodes involved in the dissemination of information in presence (resp., absence) of the virtual IoT V. Specifically, we computed $num_{st}^{V}$ by performing the information dissemination task only through its nodes; instead, we obtained $\hat{num_{st}^{V}}$ by performing the same task on the whole MIoT. Finally, we computed: $f_{st}=\frac{num_{st}^{V}}{\hat{num_{st}^{V}}}$. Clearly, the lower $f_{st}$, the higher the contribution of the virtual IoTs in reducing the number of nodes necessary for the information dissemination task and, consequently, the higher the contribution that our virtual IoT detection approach can provide to information dissemination.

We computed the average values of $f_{st}$ by operating on the ten MIoTs introduced in Section 6.2 and by performing the same tasks described therein for both the supervised and the unsupervised approaches. The obtained results are reported in Table 10 and Table 11.

From the analysis of these tables we can observe that both the supervised and the unsupervised approaches really contribute to decrease the number of the nodes of a MIoT involved in the information dissemination, and, therefore, to increase the efficiency of this task. As for the supervised approach, we observe that the decrease of the number of involved nodes is always high. It becomes very high as the MIoT size and the number of keywords composing the query increase. As for the unsupervised approach, we observe that it leads to a decrease of the number of the MIoT nodes involved in the dissemination task. However, this decrease is minimum for small MIoTs, whereas it becomes significant for large ones (i.e., for MIoTs with a number of nodes higher than 1256).

We performed a second experiment in this direction. Specifically, given a pair $\left(n_{s},n_{t}\right)$ of a MIoT such that information must be disseminated from $n_{s}$ to $n_{t}$ and there exists at least one virtual IoT V comprising both $n_{s}$ and $n_{t}$, we computed the fraction $g_{st}^{V}$ (resp., $\hat{g_{st}^{V}}$) of the nodes of the MIoT involved in the diffusion of information from $n_{s}$ to $n_{t}$ and having at least one content of the disseminated information registered in their profile (which implies that, in principle, they could benefit from the information they are required to disseminate). As in the previous experiment, we computed $g_{st}^{V}$ by assuming the existence of V and, hence, by performing the information dissemination task through it; by contrast, we computed $\hat{g_{st}^{V}}$ by carrying out the information dissemination task through the whole MIoT. Finally, we computed $g_{st}=\frac{g_{st}^{V}}{\hat{g_{st}^{V}}}$. Roughly speaking, it denotes how much the presence of the virtual IoT V can contribute to require information dissemination tasks only to nodes possibly benefiting of it. A value of this coefficient higher than 1 denotes a positive contribution of V; the higher this value the higher the contribution. As in the previous experiment, we computed the average values of $g_{st}$ by operating on the ten MIoTs introduced in Section 6.2 and by performing the same tasks described therein for both the supervised and the unsupervised approaches. The obtained results are reported in Table 12 and Table 13.

The analysis of these tables is a further confirmation of the efficiency of our approach. Indeed, thanks to the presence of virtual IoTs, the fraction of nodes participating to the spreading of information that can also benefit from this task increases remarkably.

The results of Table 10 and Table 11, along with the ones of Table 12 and Table 13, agree to evidence that the discovery of virtual IoTs is highly beneficial in terms of efficiency for the information dissemination task in a MIoT. In this case, the contribution of V in increasing the efficiency of the spreading task, by limiting it mainly to nodes that could benefit from the information they are disseminating, is very high for the supervised approach when |Q|=1 or |Q|=2. When |Q| increases, this contribution decreases, even if it remains still significant. As for the unsupervised approach, the contribution of V can be always observed even if it is less evident than the one characterizing the supervised approach.

### 6.6. Number and Size of Returned Virtual IoTs

This last experiment makes sense only for the unsupervised approach. Through it we aimed at investigating how the number and the size of returned virtual IoTs (and, therefore, the number and the size of returned clusters) vary when the MIoT size increases. To make this experiment significant, we maintained constant all the parameters of the adopted clustering algorithm. We considered the MIoTs $M_{1}\cdots M_{10}$ used in the previous experiments because, in this way, we had the possibility to investigate MIoT sizes ranging from 176 to 5024 nodes. We report the obtained results in Table 14.

From the analysis of this table we can observe that the average size of virtual IoTs:

- increases when the MIoT size ranges from 176 to 946;
- slightly increases when the MIoT size ranges from 946 to 2028;
- remains essentially constant when the MIoT size is higher than 2028.

In the meantime, the number of clusters:

- slightly increases when the MIoT size ranges from 176 to 946;
- increases when the MIoT size ranges from 946 to 2028;
- highly increases when the MIoT size is higher than 2028.

The obtained results are extremely interesting because they confirm the soundness of the reasoning made in Section 5.3. In particular, this experiment confirms the scalability of our approach. As a matter of fact, after the virtual IoTs have been constructed offline, their usage for querying and for the other tasks of interest for the user can be performed online. Now, we observed that the number of available virtual IoTs highly increases when the MIoT size increases. However, because the size of each virtual IoT is only slightly impacted by the growth of the corresponding MIoT, and because user tasks generally involve one or at most a few of available virtual IoTs, we can conclude that our approach is scalable with respect to the size variation of the MIoT.

## 7. Conclusions

In this paper, we have discussed about the attempt of “humanizing” things. We have seen that this trend will become increasingly challenging in the future because things are becoming more and more smart and social. As a consequence, it appears natural to apply concepts typical of social networking to the Internet of Things. Actually, as things are becoming increasingly heterogeneous in their formats, semantics and behaviors, it appears even better to apply social internetworking ideas and concepts to this scenario.

For this reason, we have decided to adopt the MIoT paradigm as the reference one for our proposal. With the support of this paradigm, we have proposed a rich and high-level abstraction profile of a thing, taking into account the content that it exchanged with the other things in the past. Then, we have introduced the concept of topic-guided virtual IoT and we have proposed a supervised and an unsupervised approach to constructing topic-guided virtual IoTs in a Multi-IoT scenario.

This paper must not be considered as an ending point; on the contrary, it is a starting point for future research efforts. Indeed, the definition of a thing’s profile and the usage of paradigms, like MIoT, allowing multiple IoTs to be modeled in a way analogous to how multiple social networks interacting with each other are modeled, allow us to investigate the possible extension to the IoT context of many research themes already analyzed for social networks. For instance, it could be possible: *(i)* to model the concepts of trust and reputation of a thing in the IoTs it belongs to; *(ii)* to develop “team building” approaches aiming at constructing teams of things to perform a certain activity; *(iii)* to investigate new forms of centrality of a thing in a MIoT based on both its position and its profile. Actually, these are just three of the many possible future developments of our research in such a rapidly evolving and very promising scenario.

## Figures and Tables

**Table 1:** Main features of the constructed MIoTs.

MIoT (Size)	Number of Arcs	Mean In-Degree	Mean Out-Degree	Number of i-arcs	Number of c-arcs
$M_{1}$ (176)	1176	6.29	6.61	980	126
$M_{2}$ (301)	2050	7.76	7.74	1709	341
$M_{3}$ (485)	3756	8.80	8.54	3130	626
$M_{4}$ (778)	5866	8.89	9.11	4895	971
$M_{5}$ (946)	7624	8.64	8.84	6422	1202
$M_{6}$ (1256)	9860	7.87	7.98	7917	1943
$M_{7}$ (1725)	12,263	7.94	8.18	9964	2299
$M_{8}$ (2028)	15,568	8.22	8.38	12,857	2711
$M_{9}$ (3544)	26,428	8.36	8.42	22,718	3710
$M_{10}$ (5024)	38,642	8.44	8.54	33,724	4918

**Table 2:** Values of the clustering coefficient for real and virtual IoTs against the size of MIoTs and queries used to generate the virtual IoTs (supervised approach).

MIoT (Size)	Avg. Clustering Coeff. (Real IoTs)	Avg. Clustering Coeff. (Virtual IoTs)					
		|Q|=1	|Q|=2	|Q|=4	|Q|=6	|Q|=8	|Q|=10
$M_{1}$ (176)	0.230	0.318	0.368	0.389	0.394	0.401	0.408
$M_{2}$ (301)	0.272	0.343	0.388	0.419	0.424	0.434	0.446
$M_{3}$ (485)	0.293	0.396	0.437	0.477	0.482	0.488	0.497
$M_{4}$ (778)	0.353	0.447	0.478	0.503	0.508	0.511	0.517
$M_{5}$ (946)	0.371	0.452	0.492	0.512	0.522	0.524	0.526
$M_{6}$ (1256)	0.385	0.486	0.511	0.529	0.530	0.532	0.535
$M_{7}$ (1725)	0.386	0.501	0.524	0.536	0.537	0.538	0.539
$M_{8}$ (2028)	0.388	0.519	0.536	0.541	0.541	0.542	0.543
$M_{9}$ (3544)	0.392	0.522	0.540	0.544	0.544	0.545	0.546
$M_{10}$ (5024)	0.395	0.534	0.546	0.546	0.546	0.547	0.548

**Table 3:** Values of the density for real and virtual IoTs against the size of MIoTs and queries used to generate the virtual IoTs (supervised approach).

MIoT (Size)	Average Density (Real IoTs)	Average Density (Virtual IoTs)					
		|Q|=1	|Q|=2	|Q|=4	|Q|=6	|Q|=8	|Q|=10
$M_{1}$ (176)	0.348	0.260	0.264	0.280	0.289	0.296	0.301
$M_{2}$ (301)	0.262	0.292	0.303	0.309	0.315	0.320	0.324
$M_{3}$ (485)	0.274	0.390	0.395	0.400	0.402	0.405	0.408
$M_{4}$ (778)	0.269	0.476	0.483	0.490	0.501	0.509	0.514
$M_{5}$ (946)	0.276	0.492	0.509	0.521	0.536	0.534	0.556
$M_{6}$ (1256)	0.284	0.547	0.556	0.567	0.572	0.576	0.581
$M_{7}$ (1725)	0.278	0.582	0.582	0.594	0.598	0.598	0.601
$M_{8}$ (2028)	0.273	0.609	0.610	0.620	0.626	0.630	0.639
$M_{9}$ (3544)	0.269	0.626	0.628	0.630	0.634	0.636	0.637
$M_{10}$ (5024)	0.262	0.636	0.636	0.638	0.638	0.640	0.642

**Table 4:** Values of both clustering coefficient and density of real and virtual IoTs against the size of MIoTs (unsupervised approach).

MIoT (Size)	Average Clustering Coefficient		Average Density	
	Real IoTs	Virtual IoTs	Real IoTs	Virtual IoTs
$M_{1}$ (176)	0.230	0.473	0.348	0.315
$M_{2}$ (301)	0.272	0.499	0.262	0.350
$M_{3}$ (485)	0.293	0.500	0.274	0.375
$M_{4}$ (778)	0.353	0.511	0.269	0.318
$M_{5}$ (946)	0.372	0.509	0.276	0.316
$M_{6}$ (1256)	0.385	0.506	0.284	0.314
$M_{7}$ (1725)	0.386	0.522	0.280	0.328
$M_{8}$ (2028)	0.388	0.535	0.273	0.360
$M_{9}$ (3544)	0.394	0.547	0.271	0.364
$M_{10}$ (5024)	0.398	0.562	0.269	0.368

**Table 5:** Average fraction of merged c-nodes against the size of MIoTs and queries used to generate the virtual IoTs (supervised approach).

MIoT (Size)	Average Fraction of Merged C-Nodes					
	|Q|=1	|Q|=2	|Q|=4	|Q|=6	|Q|=8	|Q|=10
$M_{1}$ (176)	0.304	0.455	0.517	0.532	0.554	0.572
$M_{2}$ (301)	0.380	0.515	0.608	0.627	0.652	0.679
$M_{3}$ (485)	0.539	0.661	0.782	0.798	0.813	0.823
$M_{4}$ (778)	0.690	0.786	0.860	0.874	0.883	0.892
$M_{5}$ (946)	0.724	0.812	0.884	0.898	0.916	0.924
$M_{6}$ (1256)	0.808	0.883	0.939	0.943	0.946	0.948
$M_{7}$ (1725)	0.862	0.908	0.952	0.961	0.961	0.963
$M_{8}$ (2028)	0.908	0.959	0.974	0.975	0.976	0.977
$M_{9}$ (3544)	0.928	0.963	0.976	0.977	0.977	0.978
$M_{10}$ (5024)	0.936	0.968	0.978	0.979	0.980	0.981

**Table 6:** Average fraction of real IoTs involved in a virtual IoT against the size of MIoTs and queries used to generate the virtual IoTs (supervised approach).

MIoT (Size)	Average Fraction of Involved Real IoTs					
	|Q|=1	|Q|=2	|Q|=4	|Q|=6	|Q|=8	|Q|=10
$M_{1}$ (176)	0.373	0.467	0.488	0.476	0.452	0.448
$M_{2}$ (301)	0.365	0.469	0.525	0.501	0.488	0.480
$M_{3}$ (485)	0.482	0.477	0.448	0.442	0.435	0.432
$M_{4}$ (778)	0.457	0.432	0.418	0.415	0.413	0.411
$M_{5}$ (946)	0.455	0.482	0.624	0.628	0.647	0.644
$M_{6}$ (1256)	0.453	0.514	0.805	0.864	0.917	0.924
$M_{7}$ (1725)	0.482	0.577	0.815	0.872	0.917	0.924
$M_{8}$ (2028)	0.514	0.672	0.833	0.898	0.917	0.924
$M_{9}$ (3544)	0.584	0.704	0.844	0.905	0.924	0.926
$M_{10}$ (5024)	0.624	0.727	0.888	0.911	0.928	0.934

**Table 7:** Average fraction of merged c-nodes and average fraction of real IoTs involved in a virtual IoT against the size of MIoTs (unsupervised approach).

MIoT (Size)	Average Fraction of Merged C-Nodes	Average Fraction of Involved Real IoTs
$M_{1}$ (176)	0.227	0.361
$M_{2}$ (301)	0.306	0.353
$M_{3}$ (485)	0.309	0.357
$M_{4}$ (778)	0.342	0.356
$M_{5}$ (946)	0.334	0.359
$M_{6}$ (1256)	0.326	0.361
$M_{7}$ (778)	0.332	0.360
$M_{8}$ (2028)	0.335	0.358
$M_{9}$ (3544)	0.341	0.371
$M_{10}$ (5024)	0.344	0.378

**Table 8:** Average Herfindahl Index of virtual IoTs against the size of MIoTs and queries used to generate the virtual IoTs (supervised approach).

MIoT (Size)	Average Herfindhal Index					
	|Q|=1	|Q|=2	|Q|=4	|Q|=6	|Q|=8	|Q|=10
$M_{1}$ (176)	0.207	0.186	0.177	0.175	0.173	0.172
$M_{2}$ (301)	0.204	0.183	0.174	0.173	0.172	0.171
$M_{3}$ (485)	0.178	0.173	0.170	0.170	0.169	0.168
$M_{4}$ (778)	0.172	0.172	0.170	0.170	0.169	0.168
$M_{5}$ (946)	0.172	0.170	0.169	0.169	0.169	0.168
$M_{6}$ (1256)	0.173	0.168	0.167	0.169	0.168	0.167
$M_{7}$ (1725)	0.170	0.168	0.167	0.169	0.168	0.167
$M_{8}$ (2028)	0.168	0.167	0.167	0.167	0.167	0.167
$M_{9}$ (3544)	0.168	0.167	0.167	0.167	0.167	0.167
$M_{10}$ (5024)	0.167	0.167	0.167	0.167	0.167	0.167

**Table 9:** Average Herfindahl Index of virtual IoTs against the size of MIoTs (unsupervised approach).

MIoT (Size)	Average Herfindahl Index
$M_{1}$ (176)	0.658
$M_{2}$ (301)	0.543
$M_{3}$ (485)	0.658
$M_{4}$ (778)	0.636
$M_{5}$ (946)	0.654
$M_{6}$ (1256)	0.694
$M_{7}$ (1725)	0.656
$M_{8}$ (2028)	0.635
$M_{9}$ (3544)	0.664
$M_{10}$ (5024)	0.686

**Table 10:** Average values of $f_{st}$ against the size of MIoTs and queries used to generate the virtual IoTs (supervised approach).

MIoT (Size)	Average $f_{\mathrm{st}}$					
	|Q|=1	|Q|=2	|Q|=4	|Q|=6	|Q|=8	|Q|=10
$M_{1}$ (176)	0.144	0.220	0.290	0.304	0.336	0.347
$M_{2}$ (301)	0.126	0.170	0.177	0.175	0.178	0.179
$M_{3}$ (485)	0.104	0.112	0.074	0.052	0.041	0.037
$M_{4}$ (778)	0.057	0.051	0.028	0.038	0.047	0.049
$M_{5}$ (946)	0.048	0.034	0.022	0.028	0.032	0.024
$M_{6}$ (1256)	0.031	0.015	0.017	0.011	0.007	0.007
$M_{7}$ (1725)	0.026	0.014	0.011	0.010	0.008	0.008
$M_{8}$ (2028)	0.016	0.010	0.009	0.009	0.009	0.009
$M_{9}$ (3544)	0.012	0.009	0.009	0.009	0.009	0.009
$M_{10}$ (5024)	0.011	0.008	0.007	0.007	0.007	0.007

**Table 11:** Average values of $f_{st}$ against the size of MIoTs (unsupervised approach).

MIoT (Size)	Average $f_{\mathrm{st}}$
$M_{1}$(176)	0.904
$M_{2}$(301)	0.722
$M_{3}$(485)	0.635
$M_{4}$(778)	0.584
$M_{5}$(946)	0.580
$M_{6}$(1256)	0.576
$M_{7}$(1725)	0.516
$M_{8}$(2028)	0.477
$M_{9}$(3544)	0.452
$M_{10}$(5024)	0.426

**Table 12:** Average values of $g_{st}$ against the size of MIoTs and queries used to generate the virtual IoTs (supervised approach).

MIoT (Size)	Average $g_{\mathrm{st}}$					
	|Q|=1	|Q|=2	|Q|=4	|Q|=6	|Q|=8	|Q|=10
$M_{1}$ (176)	4.018	2.792	2.223	1.918	1.331	1.321
$M_{2}$ (301)	3.563	2.619	2.445	2.009	1.683	1.664
$M_{3}$ (485)	3.269	2.370	1.426	1.528	1.626	1.674
$M_{4}$ (778)	3.130	2.168	2.367	1.916	1.494	1.325
$M_{5}$ (946)	3.232	2.102	1.864	1.712	1.461	1.391
$M_{6}$ (1256)	3.467	1.979	1.378	1.412	1.438	1.452
$M_{7}$ (1725)	3.476	2.224	1.414	1.444	1.494	1.492
$M_{8}$ (2028)	3.496	2.669	1.489	1.491	1.521	1.545
$M_{9}$ (3544)	3.507	2.712	1.612	1.624	1.631	1.632
$M_{10}$ (5024)	3.517	2.926	1.783	1.841	1.864	1.874

**Table 13:** Average values of $g_{st}$ against the size of MIoTs (unsupervised approach).

MIoT (Size)	Average $g_{\mathrm{st}}$
$M_{1}$ (176)	1.341
$M_{2}$ (301)	1.269
$M_{3}$ (485)	1.211
$M_{4}$ (778)	1.177
$M_{5}$ (946)	1.173
$M_{6}$ (1256)	1.171
$M_{7}$ (1725)	1.194
$M_{8}$ (2028)	1.273
$M_{9}$ (3544)	1.281
$M_{10}$ (5024)	1.301

**Table 14:** Average size and number of virtual IoTs against the increase of the MIoT size (unsupervised approach).

MIoT (Size)	Average Size of Virtual IoTs	Number of Virtual IoTs
$M_{1}$ (176)	22.44	10
$M_{2}$ (301)	28.21	13
$M_{3}$ (485)	36.64	16
$M_{4}$ (778)	40.82	22
$M_{5}$ (946)	44.66	24
$M_{6}$ (1256)	46.74	30
$M_{7}$ (1725)	48.12	39
$M_{8}$ (2028)	50.24	45
$M_{9}$ (3544)	50.46	78
$M_{10}$ (5024)	50.64	105
